# Queering medical education: systematically assessing LGBTQI health competency and implementing reform

**DOI:** 10.1080/10872981.2018.1510703

**Published:** 2018-08-29

**Authors:** Timothy DeVita, Casey Bishop, Michael Plankey

**Affiliations:** Department of Medicine, Georgetown University School of Medicine, Washington, DC, USA

**Keywords:** Medical education curriculum, LGBTQI, AAMC, assessment, cultural competency

## Abstract

Lesbian, gay, bisexual, transgender, queer, and intersex (LGBTQI) individuals face well-established health disparities. American medical schools have been inconsistent in their training in the care of LGBTQI-identified patient, and many have not formally assessed their curriculums for content related to the care of LGBTQI-identified patients. From 2015 to 2016, the authors systematically evaluated Georgetown University School of Medicine’s preclinical curriculum for its LGBTQI competency using video lecture capture, LGBTQI health competencies published by the American Association of Medical Colleges (AAMC) and learning objectives developed by Vanderbilt University. Based on the results of the curricular audit, the authors have created didactic content targeted at the identified curricular gaps that has been implemented throughout the preclinical curriculum at Georgetown. The curricular auditing process described here could be replicated at other medical schools, which would allow educators to develop targeted content to address unmet competencies.

**Abbreviations** AAMC: Association of American Medical Colleges; LGBTQI: Lesbian, Gay, Bisexual, Transgender, Questioning, Intersex

## Problem

There is a wide and expanding body of literature that documents that lesbian, gay, bisexual, transgender, queer and intersex (LGBTQI)-identified patients face various physical, behavioural, and sexual health disparities [1]. Some disparities, such as sexually transmitted infections and mental health disorders, are well known, while others such as cancer, obesity, heart disease, and homelessness are less ubiquitous. This article describes systematic methods for medical schools to audit their curriculums for LGBTQI content and implement targeted reform to fill curricular gaps.

Although the disparities LGBTQI communities face are increasingly well documented, gaps remain in the training of health care professionals to reverse these disparities. Some medical schools have emerged as leaders in the field of LGBTQI medical education, while other medical schools have limited engagement with the topic. A median of only 5 h is directed towards LGBTQI health in American medical schools [2]. A survey of 170 medical schools and 4,262 students found that most students view their school’s LGBT-curriculum as ‘fair’ or worse, with the largest gaps in gender transitioning and sex reassignment surgery [3]. Other studies echoed the sentiment that transgender care is the most lacking portion of LGBTQI healthcare in medical education [4,5]. Such a gap in medical school training has left some physicians ill equipped to meet the needs of their LGBTQI patients. In some instances, lack of medical training, along with bias, has led LGBTQI patients to fear abuse in healthcare settings [6,7]. Such abuse is echoed by LGBTQI identified medical students, almost half of which report witnessing ‘anti-LGBT jokes, rumors, and/or bullying by fellow medical students and/or other members of the healthcare team’ [8]. LGBTQI patients have reported health care professionals refusing to touch them, blaming them for their health status, using harsh/abusive language, and sometimes even being physically abusive. Occasionally, LGBTQI patients are left to explain their health needs to their physician because the physician may be poorly prepared to identify or manage their concerns.

There is a new and growing body of literature advocating physician training toward the needs of LGBTQI patients. In 2007, the American Association of Medical Colleges (AAMC) published ‘Institutional Programs and Educational Activities to Address the Needs of Gay, Lesbian, Bisexual and Transgender (GLBT) Students and Patients,’ which called for safe learning environments for LGBTQI students and staff [9]. In addition, the guidelines petitioned for medical schools to teach the ‘knowledge, skills, and attitudes that graduating medical students should possess in the area of human sexuality, including sexual orientation and gender identity, sufficient to prepare them to provide excellent, comprehensive health care to GLBT patients.’ In 2010, the American Medical Association published an article in the AMA Journal of Ethics titled, ‘The Medical School Curriculum and LGBT Health Concerns,’ which echoed the AAMC’s call for LGBTQI medical education [10].

There is also more literature proposing methods to address the issue of physician training to meet the needs of LGBTQI patients. In 2012, Vanderbilt’s Gay Straight Alliance created a set of learning objectives that were published in ‘Including LGBTQI Education at Vanderbilt University School of Medicine’ [11]. These competencies were influenced by the AAMC competencies as well as 5 medical schools that are proactive in LGBTQI health education: Brown University, University of California-Irvine, University of California-San Francisco, University of Massachusetts, and University of Pennsylvania. The Vanderbilt Gay Straight Alliance integrated the findings to create a standardized list of LGBTQI health topics in medical curricula. Because the competencies are designed for a specific medical school, they are very specific. They outline actual clinical knowledge and skills that are important for a physician to master to properly care for an LGBTQI-identified patient. In 2014, the AAMC published ‘Implementing Curricular and Institutional Climate Changes to Improve Health Care for Individuals Who are LGBT, Gender Nonconforming, or Born with DSD: A Resource for Medical Educators’ [12]. This publication included a list of 30 broad competencies across 5 curricular domains, outlining how medical schools could teach students how to care for LGBTQI patients. In 2017, the University of California–San Francisco published a report on the effectiveness innovative 8-year-old, 10-contact hour weekend elective course in LGBTQI health for health profession students [5,13]. Through these publications, the American medical community is beginning to address the health disparities that exist between each of the LGBTQI communities and the general population.

While there has been some advancement in medical training in the care of LGBTQI patients through the publishing of competency lists, literature regarding methods to assess medical school curricula and implement reform is lacking. To address the gap in this literature, medical students and leaders at the Georgetown University School of Medicine (GUSOM) have consolidated resources and created a unique method to systematically assess preclinical curricula for its LGBTQI-related content and implement targeted reform. The systematic curricular review described herein can be repeated to bring about reform in many aspects of medical education in other medical schools. The curricular audit was presented at the 2017 National Conference on Race & Ethnicity in American Higher Education (NCORE) [14].

## Audit methods

The systematic curricular audit utilizes pre-recorded video lecture captures for the ‘preclinical’ years of the curriculum. Lecture capture is an umbrella term for audio and video recordings of individual lectures taught in the preclinical classroom. The video captures the computer screen that the individual lecturers use over the course of the lecture along with the lecturer’s voice. This encompasses the progression of slides, videos, and Smart Board^TM^ drawings that are projected in the classroom in real time along with the audio content of the lecturer. Video lecture capture is a technology used by most medical schools to allow students to review the course material later, or if they are absent the day of the lecture. Because video lecture capture records almost all aspects of preclinical education, it is a particularly useful medium for curricular assessment. All of the lectures in the preclinical curriculum are recorded yearly, and can easily be manipulated in a systematic and objective study. Moreover, any medical school that uses such technology can use these methods. However, a key limitation is that it can only be used to evaluate the mostly lecture-based preclinical years of medical education, but not the clinical years.

The first phase of curricular assessment identifies what LGBTQI health content the preclinical curriculum already contains. Because the focus was on LGBTQI-related health content, medical schools can examine the video lecture captures of every lecture in the relevant course modules in the preclinical curriculum. At GUSOM, we reviewed the preclinical curriculum for its LGBTQI health content from 2015 to 2016. We focused our assessment on the following lectures and modules: an elective lecture on LGBTQI health needs, Infectious Diseases I, Infectious Diseases II, Reproductive Health, Endocrinology, and Central Nervous System II (Psychiatry).

While reviewing the Lecture captures, educators can record the existing material. At Georgetown, we used Microsoft Word to document all LGBTQI-related content that was spoken by the lecturer or written on slides as well as its timing in the lecture. Oral and written content were given equal weight. The document consolidated all LGBTQI related content existing in the preclinical curriculum at that time. At Georgetown, the document totalled 13 pages in length.

After all of the existing LGBTQI health content in the preclinical curriculum is identified, it is necessary to compare it to nationally established assessment tools. At Georgetown, the first assessment tool we used was the published LGBTQI health competencies of the AAMC. As previously mentioned, the AAMC created 30 competencies that cover 8 domains: patient care, knowledge for practice, practice-based learning and improvement, interpersonal and communication skills, professionalism, systems-based practice, interprofessional collaboration, and personal and professional development [11]. The AAMC’s competencies are especially broad, which makes them easily applicable to the diversity of medical schools in the USA. We decided to use a second assessment tool, the learning objectives of the Vanderbilt University School of Medicine, which are much more specific [10].

The second and final phase of curricular reform objectively compares existing LGBTQI curricular content to the nationally established assessment tools. For example, at Georgetown we took the Word document we created in the identification phase of our assessment, which consolidated all written and spoken LGBTQI-related health content in the GUSOM preclinical curriculum, and compared it with both the AAMC competency and Vanderbilt learning objective lists. In the comparison, educators can designate each competency/learning objective as ‘completely met,’ ‘partially met,’ or ‘unmet’ by the existing preclinical curriculum. If the all aspects of the competency/learning objective were included in the Word document, the competency is determined to be ‘completely met.’ If only some aspects of the competency/learning objective are included in the word document and other aspects were not, then it is deemed ‘partially met,’ and so on.

## Audit results at Georgetown

We found that Georgetown’s existing curriculum completely covered 7 AAMC competencies, partially covered 8 competencies, and did not cover 15 competencies. The list of the unmet, partially met, and completely met AAMC competencies can be found in Table 1. The unmet competencies tended to be in mental health and endocrinology. The fact that GUSOM does not cover half of the prescribed competencies by the AAMC makes a strong point that the information is not being presented to the students.

**Table 1.** AAMC competencies.

Met	Sensitively and effectively eliciting relevant information about sex anatomy, sex development, sexual behavior, sexual history, sexual orientation, sexual identity, and gender identity from all patients in a developmentally appropriate manner.
	Understanding typical (male and female) sex development and knowing the main etiologies of atypical sex development.
	Developing rapport with all individuals (patient, families, and/or members of the health care team) regardless of others’ gender identities, gender expressions, body types, sexual identities, or sexual orientations, to promote respectful and affirming interpersonal exchanges, including by staying current with evolving terminology.
	Recognizing and respecting the sensitivity of certain clinical information pertaining to the care of the patient populations described above, and involving the patient (or the guardian of a pediatric patient) in the decision of when and how to communicate such information to others.
	Recognizing the unique aspects of confidentiality regarding gender, sex, and sexuality issues, especially for the patients described above, across the developmental spectrum, and by employing appropriate consent and assent practices.
	Valuing the importance of interprofessional communication and collaboration in providing culturally competent, patient-centered care to the individuals described above and participating effectively as a member of an interdisciplinary health care team.
	Identifying important clinical questions as they emerge in the context of caring for the individuals described above, and using technology to find evidence from scientific studies in the literature and/or existing clinical guidelines to inform clinical decision making and improve health outcomes.
Partially Met	Performing a complete and accurate physical exam with sensitivity to issues specific to the individuals described above at stages across the lifespan. This includes knowing when particulars of the exam are essential and when they may be unnecessarily traumatizing (as may be the case, for example, with repeated genital exams by multiple providers)
	Recognizing the unique health risks and challenges often encountered by the individuals described above, as well as their resources, and tailoring health messages and counseling efforts to boost resilience and reduce high-risk behaviors.
	Providing effective primary care and anticipatory guidance by utilizing screening tests, preventive interventions, and health care maintenance for the populations described above (e.g., screening all individuals for inter-partner violence and abuse; assessing suicide risk in all youth who are gender nonconforming and/or identify as gay, lesbian, bisexual and/or transgender; and conducting screenings for transgender patients as appropriate to each patient’s anatomical, physiological, and behavioral histories).
	Defining and describing the differences among: sex and gender; gender expression and gender identity; gender discordance, gender nonconformity, and gender dysphoria; and sexual orientation, sexual identity, and sexual behavior.
	Identifying communication patterns in the health care setting that may adversely affect care of the described populations, and learning to effectively address those situations in order to protect patients from the harmful effects of implicit bias or acts of discrimination.
	Critically recognizing, assessing, and developing strategies to mitigate one’s own implicit (i.e., automatic or unconscious) biases in providing care to the individuals described above and recognizing the contribution of bias to increased iatrogenic risk and health disparities.
	Understanding and addressing the special challenges faced by health professionals who identify with one or more of the populations described above in order to advance a health care environment that promotes the use of policies that eliminate disparities (e.g., employee nondiscrimination policies, comprehensive domestic partner benefits, etc.).
Unmet	Describing the special health care needs and available options for quality care for transgender patients and for patients born with DSD (e.g., specialist counseling, pubertal suppression, elective and nonelective hormone therapies, elective and nonelective surgeries, etc.)
	Understanding and explaining how stages of physical and identity development across the lifespan affect the above-described populations and how health care needs and clinical practice are affected by these processes.
	Understanding and describing historical, political, institutional, and sociocultural factors that may underlie health care disparities experienced by the populations described above.
	Recognizing the gaps in scientific knowledge (e.g., efficacy of various interventions for DSD in childhood; efficacy of various interventions for gender dysphoria in childhood) and identifying various harmful practices (e.g., historical practice of using ‘reparative’ therapy to attempt to change sexual orientation; withholding hormone therapy from transgender individuals) that perpetuate the health disparities for patients in the populations described above.
	Critically recognizing, assessing, and developing strategies to mitigate the inherent power imbalance between physician and patient or between physician and parent/guardian, and recognizing how this imbalance may negatively affect the clinical encounter and health care outcomes for the individuals described above.
	Demonstrating the ability to elicit feedback from the individuals described above about their experience in health care systems and with practitioners, and identifying opportunities to incorporate this feedback as a means to improve care (e.g., modification of intake forms, providing access to single-stall, gender-neutral bathrooms, etc.)
	Understanding that implicit (i.e., automatic or unconscious) bias and assumptions about sexuality, gender, and sex anatomy may adversely affect verbal, nonverbal, and/or written communication strategies involved in patient care, and engaging in effective corrective self- reflection processes to mitigate those effects.
	Recognizing and sensitively addressing all patients’ and families’ healing traditions and beliefs, including health-related beliefs, and understanding how these might shape reactions to diverse forms of sexuality, sexual behavior, sexual orientation, gender identity, gender expression, and sex development.
	Accepting shared responsibility for eliminating disparities, overt bias (e.g., discrimination), and developing policies and procedures that respect all patients’ rights to self-determination
	Explaining and demonstrating how to navigate the special legal and policy issues (e.g., insurance limitations, lack of partner benefits, visitation and nondiscrimination policies, discrimination against children of same-sex parents, school bullying policies) encountered by the populations described above.
	Identifying and appropriately using special resources available to support the health of the individuals described above (e.g., targeted smoking cessation programs, substance abuse treatment, and psychological support).
	Explaining how homophobia, transphobia, heterosexism, and sexism affect health care inequalities, costs, and outcomes.
	Describing strategies that can be used to enact reform within existing health care institutions to improve care to the populations described above, such as forming an LGBT support network, revising outdated nondiscrimination and employee benefits policies, developing dedicated care teams to work with patients who were born with DSD, etc.
	Demonstrating the ability to perform an appropriate risk/benefit analysis for interventions where evidence-based practice is lacking, such as when assisting families with children born with some forms of DSD, families with prepubertal gender nonconforming children, or families with pubertal gender nonconforming adolescents.

The second survey tool, the learning objective list created by Vanderbilt University, confirmed the curricular gaps determined by the first survey tool. Georgetown completely met 12 Vanderbilt learning objectives completely, partially met 7, and did not meet 13. The list of the unmet, partially met, and completely met Vanderbilt learning objectives can be found in Table 2. In general, the biggest gaps were in mental health, pediatric/geriatric LGBTQI health, and gender-affirming care for transgender patients. The consistency of the results from the 2 survey tools created by reputable organizations, the AAMC and Vanderbilt, provides solid evidence that GUSOM did not adequately prepare its students to meet the health needs of their future LGBTQI patients. The results echoed the results of similar studies, which found gaps in the care of transgender patients [4,5]. It indicated that additional learning objectives and educational content must be added to the preclinical curriculum to better prepare students to care for LGBTQI patients.

**Table 2.** Vanderbilt learning objectives.

Met	Communication/Interview Skills
	Intake Forms (gender identity, sexual orientation, relationship status, parentage)
	PBL Integration
	Embryology – Gender vs. Sex
	STIs in lesbians
	STI recommendations in MSM
	HIV in MSM
	Exclusive WSWs: Paps, Breast Exams, and HPV Screening
	MSMs and need of HepA/HPV shot
	Lesbian Obesity
	Increased heart disease rate in lesbians
	Eating disorders in MSM
Partially Met	Assumptions/Biases
	Substance Abuse Screening
	Standardized Patient Cases
	Embryology—Changing Terminology
	Anal Paps
	Anal Cancer Risks, Tx, Anal Pap in MSM
	Lesbian nulliparity and risk of breast/ovarian/cervical cancer
Unmet	Depression screening
	Embryology—Disorders of Sex Development (What are they? What are the current thoughts on treatment options? What are gender assignments?)
	Vaginitis spread in lesbians
	Availability/Efficacy of Rectal Microbicides
	Hormone Therapy Pharmacology
	Transitioning options and associated risks
	Puberty suppression in management of trans youth
	Gay teen issues (psychological/sexual/coming out/identity development/schooling)
	Gay couples and fertility options
	Gender dysphoria vs. transgender
	Depression and Suicide Rates in LGBTQI teens/adults
	LGBTQI patients and having children (medical options and legal concerns)
	LGBT Teen Issues

## Focused curricular reform

Medical schools can use the results of the systematic LGBTQI health audit to create targeted curricular reform. From the ‘partially met’ and ‘unmet’ Vanderbilt learning objectives and AAMC competencies, educators know what material must be added to the preclinical curriculum to fill in the gaps. Using this information, educators can create curricular reform targeting those deficiencies.

While medical schools may take different targeted approaches at curricular reform, at Georgetown we created a slide deck with each slide fulfilling the Vanderbilt LGBTQI learning objectives deemed to be ‘unmet’ or ‘partially met’ by the Georgetown curriculum. We decided to use the Vanderbilt learning objectives because the AAMC’s competencies lack specificity; the competencies are long, vague, and do not explicitly state specific clinical skills that should be mastered by medical students. We compared the AAMC competencies and Vanderbilt learning objectives in Figure 1. It visually maps the two, showing that the Vanderbilt learning objective list covers very similar subject matter to the AAMC competencies but in a form that states specific clinical skills. All AAMC competencies overlapped with Vanderbilt competencies, and most of the Vanderbilt competencies covered portions of 4 different AAMC competencies. Thus, we can be sure that all of the AAMC competencies will be filled by writing course content for ‘unmet’ or ‘partially met’ Vanderbilt course objectives.

**Figure 1.**
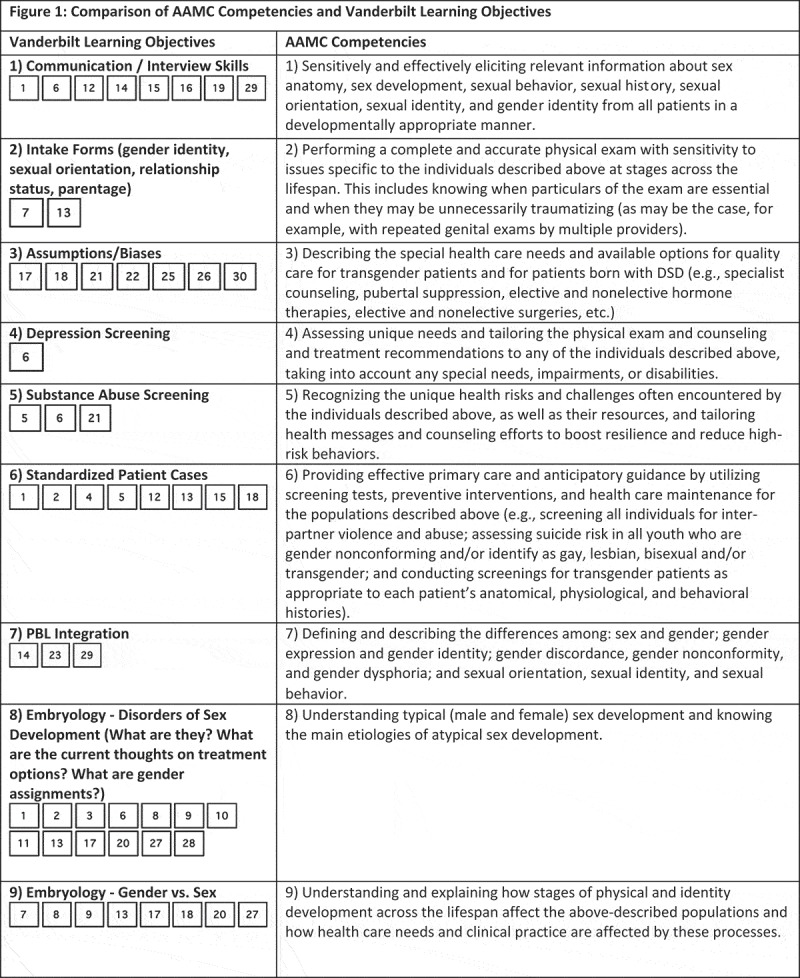
Comparison of AAMC competencies and Vanderbilt learning objectives.

**Figure 1.**
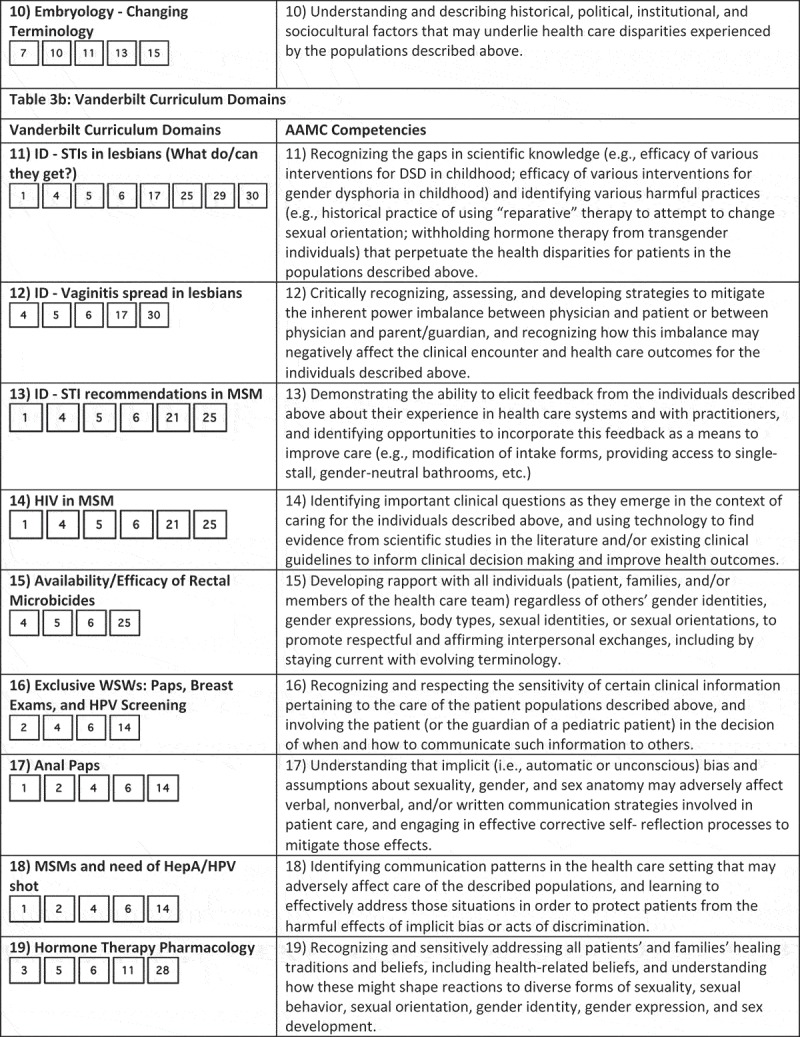
Continue.

**Figure 1.**
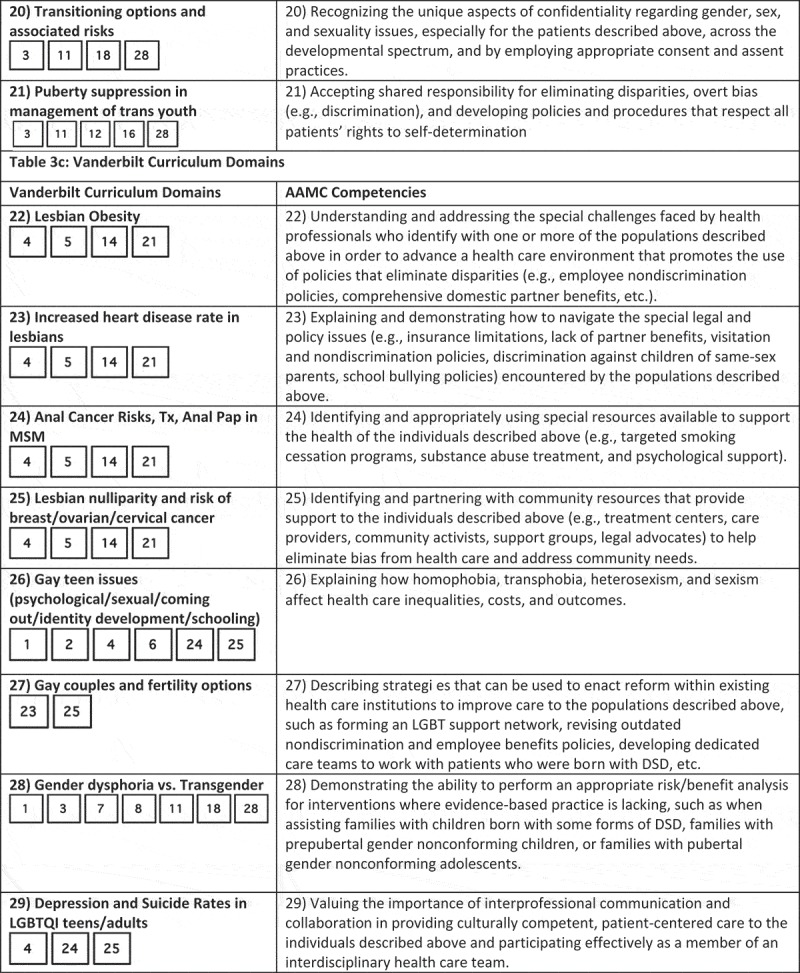
Continue.

**Figure 1.**
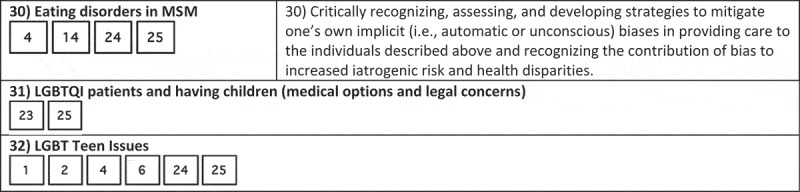
Continue.

Each slide in the deck corresponded to a ‘unmet’ or ‘partially met’ competency, so most of the deck addressed LGBTQI mental health, pediatric/geriatric LGBTQI health, or gender-affirming care for transgender patients. The deck, now approved for implementation by the School of Medicine Committee on Medical Education, has been broken down and disseminated into the various courses and lectures to which they fit into the GUSOM preclinical curriculum. We determined that integration, rather than a separate LGBT health lecture, would normalize the content and avoid the content being seen as separate or supplementary. The LGBTQI health content will be presented in a similar manner to other health disparities in the curriculum, such as gender, race, ethnicity, or socioeconomic status, giving it equal value. We disseminated the slides across the following courses: Infectious Diseases, Reproduction, Endocrinology, and Central Nervous System I & II. Examination questions will be created to incentivize and test mastery in the content.

Because the modified curriculum will be fully implemented for the GUSOM Class of 2021, which began in the Fall of 2017, the changes have yet to be validated. When the class finishes its preclinical curriculum in the spring of 2019, we plan to validate the curricular additions by examining student performance on the corresponding examination questions.

## Conclusions

Although there is a growing body of literature documenting health disparities faced by LGBTQI communities, many medical schools have not formally audited their curriculum to determine which competencies are unmet. In this way, LGBTQI health education is inconsistent in American medical schools, and some medical students are not prepared to meet the needs of their LGBTQI patients. The curricular auditing process delineated in this article from Georgetown University School of Medicine (GUSOM) is a model that other medical schools can adapt to systematically assess curriculum, compare it to national LGBTQI learning objective, and develop targeted curricular content to address unmet competencies.
